# Immunosuppressive Role of Myeloid-Derived Suppressor Cells and Therapeutic Targeting in Lung Cancer

**DOI:** 10.1155/2018/6319649

**Published:** 2018-03-25

**Authors:** Jie Ma, Huaxi Xu, Shengjun Wang

**Affiliations:** ^1^Department of Laboratory Medicine, The Affiliated People's Hospital, Jiangsu University, Zhenjiang, China; ^2^Institute of Laboratory Medicine, Jiangsu Key Laboratory of Laboratory Medicine, Jiangsu University School of Medicine, Zhenjiang, China

## Abstract

Lung cancer is the leading cause of cancer death worldwide due to its late diagnosis and poor outcome. Immunotherapy is becoming more and more encouraging and promising in lung cancer therapy. Myeloid-derived suppressor cells (MDSCs) are the main tumor suppressor factors, and the treatment strategy of targeting MDSCs is gradually emerging. In this review, we summarize what is currently known about the role of MDSCs in lung cancer. In view of the emerging importance of MDSCs in lung cancer, the treatment of targeting MDSCs will be useful to the control of the development and progression of lung cancer. However, the occurrence, metastasis, and survival of cancer is the result of multiple factors and multiple mechanisms, so combined treatments using different strategies will become the major therapy method for lung cancer in the future.

## 1. Introduction

Lung cancer is a challenging health problem and the leading cause of cancer-related mortality in developed countries, where more than 1.0 million people die of the disease each year [1]. Despite advances in the treatment of lung cancer with chemotherapy and the integration of targeted therapy, the overall outcomes remain poor. A better understanding of the immunologic properties of lung cancer has led to novel treatment strategies, including immune checkpoint modulation and vaccine therapy [2]. Recent clinical trials in lung cancer demonstrate the potential of immunotherapeutics to increase the overall survival in patients with lung cancer compared to the current standard of care [3].

Myeloid-derived suppressor cells (MDSCs) are a heterogeneous population of cells that consists of myeloid progenitor cells and immature granulocytes, immature macrophages, and immature dendritic cells (DCs) [4]. MDSCs play a critical role in tumor-associated immunosuppressive function, which plays an important role in the effective immunotherapies for cancer. In mice, MDSCs are identified by the expression of CD11b and Gr-1 on the cell surface, and the Gr-1 molecule includes Ly6G and Ly6C. CD11b^+^Ly6G^−^Ly6C^high^ cells showing monocytic-like morphology are called monocytic MDSCs (M-MDSCs), and CD11b^+^Ly6G^+^Ly6C^low^ cells showing granulocyte-like morphology are called granulocytic MDSCs (G-MDSCs) [5]. MDSCs also express histamine and histamine receptor 1 (HR1), which enhances the survival and expansion of MDSCs [6]. In humans, MDSCs are defined by the expression of CD33 on the cell surface but lack the expression of markers of mature myeloid and lymphoid cells [4]. The equivalents to PMN-MDSCs are defined as CD11b^+^CD14^−^CD15^+^ or CD11b^+^CD14^−^CD66b^+^, and equivalents to M-MDSCs, as CD11b^+^CD14^+^HLA-DR^−/low^CD15^−^ in human peripheral blood mononuclear cells (PBMC) [7]. In addition, there is a third population of MDSCs in humans. The early-stage MDSCs are termed Lin^−^HLA-DR^−^CD33^+^ [7, 8]. In cancer patients, MDSCs could strongly inhibit the antitumor immune responses of CD4^+^ T cells, CD8^+^ T cells, and NK cells and promote the progression of tumors. Currently, strategies to target MDSCs in cancer immunotherapy mainly involve promoting the differentiation of MDSCs, inhibiting their suppressive effect, or eliminating the cells.

## 2. Mechanisms of MDSC-Mediated Immune Suppression

MDSCs comprise a heterogeneous population of immature myeloid cells that exert the protumor immune response function via a variety of mechanisms. It is believed that MDSCs are major contributors to mediating tumor escapes. MDSCs are able to induce tolerance to a variety of immune responses mediated by effector T cells and NK cells. Both M-MDSCs and G-MDSCs could inhibit effector T cells by different manners [4]. M-MDSCs predominantly play the role of immune suppressor by the production of Arg-1 and generation of NO, whereas G-MDSCs mainly produce ROS and Arg-1 [8].

### 2.1. Arg-1 and NO

MDSCs are able to express high levels of Arg-1 and NO, while these two molecules have the effect of inhibiting the function of T cells [9, 10]. The suppressive activity of Arg-1 is based on its role in the hepatic urea cycle, metabolizing L-arginine to L-ornithine. A study showed that Arg-1 was closely related to the proliferation of T cells [11]. A PEGylated form of the catabolic enzyme arginase-1 (peg-Arg-1) can enhance the growth of tumors in mice in a manner that correlated with higher MDSC numbers [12]. The enhancement of the activity of Arg-1 in MDSCs causes the decomposition of arginine, which leads to the decrease of L-arginine, and inhibits the proliferation of T cells by various mechanisms, including the downregulation of CD3 expression and the inhibition of cyclin D3 and cyclin-dependent kinase 4 expression [13]. NO can inhibit the function of JAK3 and STAT5 by inducing the apoptosis of T cells [14] or inhibit the proliferation of T cells by inhibiting the expression of MHC-II [15].

### 2.2. ROS

Another important factor associated with the immunosuppressive ability of MDSCs is reactive oxygen species (ROS). The upregulation of the expression of ROS in tumor-bearing mice and tumor patients is a major feature of MDSCs [16–19]. The expression of ROS in tumor-bearing mice and tumor patients could significantly enhance the immunosuppression of MDSCs [16]. Interestingly, the binding of integrin on the surface of MDSCs after the action between MDSCs and T cells increased the expression of ROS [20]. In addition, other factors such as GM-CSF, IL-10, TGF-*β*, IL-6, PDGF, and IL-3 can induce the production of ROS by MDSCs [21].

### 2.3. Peroxynitrite

The product of the superoxide anion and NO chemical reaction is another factor that inhibits effector T cells [20]. The expression level of peroxynitrite was significantly increased in the accumulation of MDSCs and inflammatory cells. In many different tumors, a high content of peroxynitrite is closely related to the process of tumor growth. In addition, this is related to the failure of T cells to respond [22–26]. Peroxynitrite can damage the expression of MHC-II and mediate T-cell apoptosis [4, 27]. Moreover, peroxynitrite leads to the nitration of tyrosines in the TCR-CD8 complex, which can damage the conformational flexibility of the complex, affecting its interaction with peptide-loaded MHC-I and leading to the unresponsiveness of CD8^+^ T cells to antigen-specific stimulation [4, 27, 28]. In addition to inhibiting the activation of T cells, MDSCs were able to influence the immune response by interfering with the innate immune response, mainly through the influence of NK cells, macrophages, and NKT cells. The effect of MDSCs on NK cells is complex. Some subsets can inhibit the killing of NK cells by blocking the production of IFN-*γ*. Other subsets can activate NK cells and enhance the killing of them by expressing RAE-1, which interacts with NKG2D on the surface of NK cells [29, 30]. A recent report showed that IL-13 mediated the effect through the IL-4R-STAT6 pathway and induced TGF-*β*-producing CD11b^+^Gr-1^+^ MDSCs. The production of TGF-*β*, IL-13, and IL-4 impaired the function of NK cells [31].

### 2.4. Tregs

The population of regulatory T cells (Tregs) plays a crucial role in tumor immune escape [32, 33]. It has been reported that MDSCs could promote the development of Tregs [32, 33]. MDSCs have been shown to not be involved in the induction of Tregs; however, they may be involved in the differentiation of Tregs by releasing cytokines or cell-cell contact [34].

### 2.5. Exosomes

Exosomes are present in high abundance in the tumor microenvironment, where they transfer information between different cells. Deng et al. found that MDSC-derived exosomes polarize macrophages toward a tumor-promoting phenotype, demonstrating that some of the tumor-promoting functions of MDSC are mediated by MDSC-shed exosomes [35].

### 2.6. Metabolic Regulation

It has been noticed that MDSCs from tumors have a stronger immunosuppressive function than MDSCs in the peripheral lymphoid organs. Some newer studies suggest that MDSC maturation and function is under the control of metabolism in the tumor microenvironment [36, 37]. Compared to spleen-MDSCs, tumor-infiltrating MDSCs (T-MDSCs) increased fatty acid uptake and activated fatty acid oxidation (FAO) [38]. Husain et al. provide evidence of an immunosuppressive role of tumor-derived lactate in inhibiting innate immune responses against developing tumors via the regulation of MDSC activity [39]. In addition to the effects of lipid metabolism and lactate, the glycolysis pathway can also affect the maturation and function of MDSCs. Liu et al. showed that the SIRT1-mTOR/HIF-1*α* glycolytic pathway was determined by the differentiation of MDSCs [40]. mTORC1 intrinsically controls CD11b^+^Ly6C^high^ M-MDSC maturation and function by mediating cellular glycolysis activity [36].

## 3. Potential Importance of MDSCs in Lung Cancer

MDSCs may provide predictive and prognostic information in lung cancer patients. The function of MDSCs as biomarkers of lung cancer involves measurements of different cell subsets in the peripheral blood of patients. Tian et al. demonstrated that the number and frequency of peripheral CD14^+^HLA-DR^−/low^ MDSCs were significantly increased in SCLC patients compared with those in controls and the frequency of MDSCs correlated with tumor stage [41]. Two years before that, Huang et al. reported similar results in that both the frequency and absolute number of CD14^+^HLA-DR^−/low^ cells were significantly increased in the peripheral blood of NSCLC patients and indicated an association with metastasis, response to chemotherapy, and progression-free survival [42]. Expecting that the frequency and number of MDSCs could distinguish between lung cancer patients and healthy controls, immunoglobulin-like transcript 3 (ILT3), which is expressed by MDSCs [43], and arginase-1 (Arg-1) mRNA [44], which is expressed by MDSCs, could also be used as surrogate markers for the frequency of MDSCs in PBMC and as attractive targets for immune intervention. Patients with NSCLC had a significantly higher ratio of CD11b^+^CD14^+^ cells than healthy subjects, which was correlated with poor performance status and poor response to chemotherapy [45]. In a study by Zhang et al., the clinical data analysis indicated that a higher frequency of B7^−^H3^+^ MDSCs was associated with reduced recurrence-free survival in patients with NSCLC [46]. The data provide evidence that increased percentages of new M-MDSC subpopulations in advanced NSCLC patients are associated with an unfavorable clinical outcome [47].

Based on clinical experience, the treatment appears to influence levels of MDSCs in lung cancer patients. Wang et al. showed that in 20 patients with advanced NSCLC who received systemic chemotherapy, 9 partial remission cases had MDSC percentages that significantly decreased, 3 stable disease cases remained invariable, and 8 progressive disease cases had MDSC percentages that significantly increased [48]. Recently, results showed that three cycles of bevacizumab-containing regimens significantly reduced the percentage of granulocytic MDSCs compared with nonbevacizumab-based regimens [49]. Elevated serum levels of TNF-*α*, CCL-2, and CCL-4 associated with an increased NO production in circulating MDSCs might be an early indicator of incomplete radiofrequency ablation and, subsequently, a potential tumor relapse in NSCLC [50]. Taken together, the reduced levels of MDSCs may be interrelated with the efficacy of chemotherapy.

Murine models are regularly used to study the relationship between MDSCs and lung cancer. In murine models of lung cancer, B7^−^H3^+^ MDSCs were found only in the tumor microenvironment, and their frequencies increased during tumor progression [46]. Parallel increases in the level of galectin-3 with the number of MDSCs in vivo were detected after cisplatin treatment [51]. Furthermore, acute exposure to single-walled carbon nanotubes (SWCNT) induced the recruitment and accumulation of lung-associated MDSCs and the MDSC-derived production of TGF-*β*, resulting in an upregulated tumor burden in the lung [52]. As we know, smoking is the leading cause of lung cancer. The effect of smoking on MDSCs function is less reported. When Ortiz et al. exposed mice to cigarette smoke (CS) alone, it resulted in a significant accumulation in various organs of cells with typical MDSC phenotype, but these cells lacked immunosuppressive activity. When CS was combined with a single dose of urethane, it led to Gr-1^+^CD11b^+^ cells accumulating in the spleen and lung, and they had potent immunosuppressive activity [53].

MDSCs have shown an increasing trend in lung cancer patients and murine models, correlating with tumor progression, increased severity of the disease, and poor prognosis and survival.

## 4. MDSCs Are a Potential Target for Therapeutic Development in Lung Cancer

Along with the development of MDSCs, many factors have been found to regulate MDSCs in recent years. Multiple signaling pathways and cytokines were found to participate in the regulation of MDSCs. Most of the factors regulate the differentiation and maturation of myeloid cells by the JAK-STAT and NF-*κ*B signaling pathways and then affect the production and activation of MDSCs. The interaction of all these factors constitutes a complex network control system that regulates the generation and function of MDSCs.

**Figure 1:**
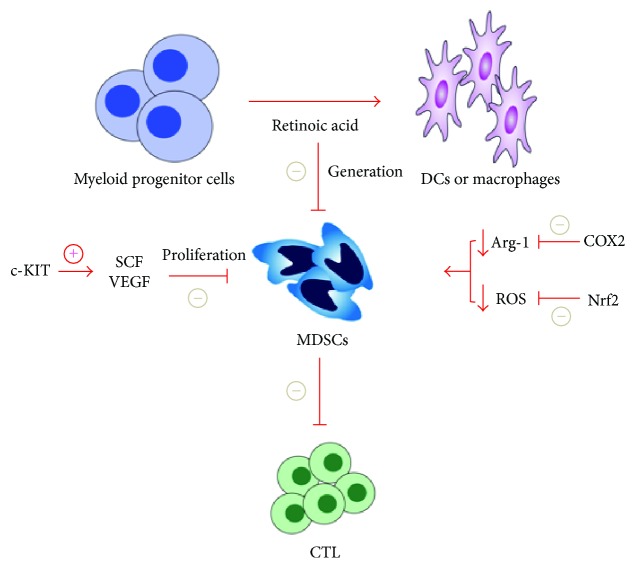
Targeting MDSCs in the treatment of lung cancer. Retinoic acid can stimulate the differentiation of myeloid progenitor cells to dendritic cells or macrophages, thereby inhibiting the differentiation of MDSCs. c-KIT can inhibit the signaling pathway which is mediated by SCF, inhibiting the amplification of MDSCs. The inhibition of COX2 expression in MDSCs can decrease the release of arginine-1, Nrf2 contributes to the clearance of ROS in MDSCs, and both COX2 and Nrf2 can inhibit the function of MDSCs. The above measures will inhibit MDSCs' immunosuppressive function on effector T cells and enhance the antitumor immunity. SCF: stem cell factor; VEGF: vascular endothelial growth factor; COX2: cyclooxygenase 2; Arg-1: arginine-1; Nrf2: nuclear factor erythroid 2 p45-related factor 2; ROS: reactive oxygen species; CTL: cytotoxic lymphocyte.

To succeed in the implementation of tumor immunotherapy, the tumor suppressor factors must be removed. MDSCs are the main tumor suppressor factors, so the treatment strategy of targeting MDSCs is gradually emerging (Figure 1). The tumor immunotherapy can be effectively enhanced by targeting the numbers and function of MDSCs. In general, the mechanisms that can be implemented to reverse the number and function of MDSCs focus on four main categories (Table 1).

### 4.1. Promotion of Myeloid Cell Differentiation

One of the most popular methods in treatment by targeting MDSCs is to promote the differentiation of immature MDSCs into myeloid cells. Retinoic acid, a product of the metabolism of vitamin A, can stimulate the differentiation of myeloid progenitor cells to dendritic cells or macrophages [54]. In a clinical trial of patients with extensive stage small cell lung cancer (SCLC), Iclozan et al. showed that vaccine alone did not affect the proportion of MDSCs, whereas in patients treated with vaccination in combination with MDSCs targeted by therapy with all-*trans*-retinoic acid (ATRA), the MDSCs decreased more than twofold [61]. In our previous study, we found that whole *β*-glucan particles (WGP) could promote the differentiation and maturation of MDSCs via the dectin-1 pathway in vitro and decrease the suppressive function of cells, thus leading to enhanced antitumor immune responses [55].

### 4.2. Inhibition of MDSC Expansion

The amplification of MDSCs is regulated by many factors, such as stem cell factor (SCF) and vascular endothelial growth factor (VEGF). c-KIT, the receptor of SCF, can inhibit the signaling pathway which is mediated by SCF, thus inhibiting the amplification of MDSCs and tumor angiogenesis [58]. VEGF is another factor that can promote the expansion of MDSCs, so it can be used as another effective target of MDSCs. However, the mechanism of VEGF in lung cancer has not been reported yet.

The STAT family, especially STAT3, plays an essential role in the regulation of the production, amplification, and function of MDSCs. STAT3 is, controversially, the main transcription factor which regulates the expansion of MDSCs. MDSCs from tumor-bearing mice have greatly increased levels of phosphorylated STAT3 compared with IMCs from naive mice, probably by upregulating the expression of STAT3 target genes, including B-cell lymphoma XL (BCL-XL), Myc, cyclin D1, and survivin. Blocking STAT3 expression in conditional knockout mice or STAT3 inhibitors could markedly reduce the expansion of MDSCs and increase T-cell responses in tumor-bearing mice [63, 64]. STAT3 can also regulate MDSCs' expansion by inducing the expression of S100A8 and S100A9 proteins, which belong to the family of S100 calcium-binding proteins that have been reported to have an important role in inflammation [65]. Wu et al. verified that Stat3C promotes MDSCs' expansion and immune suppression during lung tumorigenesis [66]. A recent report suggested that the activation of STAT3 in MDSCs and macrophages promoted tumorigenesis through pulmonary recruitment and increased resistance of suppressive cells to CD8^+^ T cells in lung cancer development [67]. The upregulation of CD45 tyrosine phosphatase activity in MDSCs exposed to hypoxia in a tumor site was responsible for the downregulation of STAT3, and STAT3 has a unique function in the tumor environment in controlling the differentiation of MDSCs into TAM [68]. In conclusion, the STAT regulatory pathway could be a potential target for lung cancer therapy.

### 4.3. Elimination of MDSCs

MDSCs can be directly eliminated in pathological settings by using some chemotherapeutic drugs and antibodies. The administration of gemcitabine to tumor-bearing mice resulted in a dramatic reduction in the number of MDSCs in the spleen and resulted in a great improvement in the antitumor response induced by immunotherapy [29]. Not only that, a combination treatment with gemcitabine and a superoxide dismutase mimetic that targets MDSCs in the tumor microenvironment can enhance the quantity and quality of both effector and memory CD8^+^ T-cell responses [69]. In addition, the generalized depletion of MDSCs, as obtained with anti-Gr-1 or anti-Ly6G antibodies, not only improves APCs, NK, and T-cell immune activities but also promotes angiostasis, leading to more efficient control of tumor growth [59].

### 4.4. Attenuation of MDSC Function

In addition, targeting MDSCs' function will be useful for controlling cancer growth and may be more efficient in combination with other immunomodulatory strategies [70]. The infiltration of MDSCs into the spleen of tumor-bearing mice was significantly decreased after being treated with gemcitabine, and the antitumor immune response was significantly enhanced [29, 71]. A recent publication reported that the treatment of mice bearing the LP07 lung adenocarcinoma with indomethacin (IND) inhibited the suppressive activity of splenic MDSCs, which restrained tumor growth through mechanisms involving CD8^+^ T cells [60]. Hoeppner et al. demonstrated that D_2_R agonists may reduce lung tumor growth through the inhibition of immunosuppressive MDSCs as well as the abrogation of tumor angiogenesis [72]. Moreover, in our previous study, we found that the inhibition of miR-9 promoted the differentiation of MDSCs with significantly reduced immunosuppressive function, whereas the overexpression of miR-9 markedly enhanced the function of MDSCs. Notably, the knockdown of miR-9 significantly impaired the activity of MDSCs and inhibited the tumor growth of Lewis lung carcinoma in mice [55].

Cyclooxygenase 2 (COX2) is considered to be a potential target molecule. The inhibition of COX2 expression in MDSCs can decrease the release of arginine, thus promoting the antitumor immune response and enhancing the effect of immune therapy. The COX2 overexpression in lung cancer and the process of epithelial mesenchymal transition (EMT) are supposed to play an important role in the inhibition of antitumor immune response by MDSCs [73, 74]. The inhibitor of ROS can also effectively reduce the immunosuppression mediated by MDSCs. In a recent study, the results indicate that the antioxidant systems directed by Nrf2 and selenoenzymes contribute to the clearance of ROS in MDSCs, efficiently preventing cancer cell metastasis [42, 75]. Zheng et al. found that cimetidine reduced MDSCs accumulating in the spleen, blood, and tumor tissue of tumor-bearing mice. Further investigation demonstrated that the NO production and Arg-1 expression of MDSCs were reduced, and MDSCs were prone to apoptosis by cimetidine treatment [76].

### 4.5. Blockade of Immune Checkpoint

In addition to the above, immune checkpoint inhibition is a new treatment approach that is undergoing extensive investigation in lung cancer. There is emerging evidence that signaling through programmed death-ligand 1 (PD-L1) plays an essential role in the immune escape of cancers linked to MDSCs [77]. Ajona et al. put forward that the combined blockade of PD-1/PD-L1 and C5a can restore antitumor immune responses, inhibit tumor cell growth, and improve outcomes of patients with lung cancer. This effect is accompanied by a negative association between the frequency of CD8 T cells and MDSCs within tumors [78]. After that, Ballbach et al. demonstrated that PD-L1 is expressed on granulocytic MDSCs upon coculture with T cells. Targeting PD-L1 also partially impaired MDSC-mediated T-cell suppression [79].

## 5. Concluding Remarks

In conclusion, MDSCs play an important role in the development and progression of lung cancer and can be used as a potential target for lung cancer treatment. We believe MDSC-targeted immunotherapy has good potential in the future. Of course, just relying on MDSCs is not enough, and the combination of different strategies should be considered, such as CAR-T immunotherapy [80, 81]. Currently, relatively new areas of research are mainly focused on the regulation of noncoding RNA in MDSCs [82] and the impact of changes in the metabolic status of MDSCs on its aggregation, differentiation, and function, which means the implications for metabolic reprogramming exist as a cancer therapeutic approach. An in-depth study of MDSCs immunotherapy will progress the treatment of lung cancer into a new era.

## Figures and Tables

**Table 1:** Regulation of MDSCs in lung cancer.

Treatment	Mouse model versus patients	Effect on MDSCs	References
Gemcitabine	Mouse models	Inhibition of MDSC expansion	[29]
Retinoic acid	Mouse models	Promotion of the differentiation of immature MDSCs	[54]
WGP	Mouse models	Promotion of the differentiation and maturation of MDSCs	[55]
Cyclooxygenase 2 inhibitor	Mouse models	Inhibition of the suppressive effects of MDSCs	[11, 56]
CSF-1 receptor antagonist(GW2580)	Mouse models	Inhibition of the recruitment of MDSCs	[57]
Anti-c-KIT	Mouse models	Inhibition of the amplification of MDSCs	[58]
Anti-Gr-1 or anti-Ly6G antibodies	Mouse models	Depletion of MDSCs	[59]
Indomethacin	Mouse models	Inhibition of the suppressive effects of MDSCs	[60]
ATRA	Patients	Inhibition of MDSCs expansion	[61]
Triterpenoids	Mouse models and patients	Inhibition of the suppressive effects of MDSCs	[62]

## Abbreviations

MDSC: Myeloid-derived suppressor cell
M-MDSC: Monocytic MDSC
G-MDSC: Granulocytic MDSC
DC: Dendritic cell
HR1: Histamine receptor 1
BCL-XL: B-cell lymphoma XL
TLR: Toll-like receptor
ADAM: A disintegrin and metalloproteinase
miRNA: MicroRNAs
ROS: Reactive oxygen species
MEF2C: Myeloid enhancement factor 2C
Arg-1: Arginase-1
Treg: Regulatory T cell
SCLC: Small cell lung cancer
NSCLC: Nonsmall cell lung cancer
SWCNT: Single-walled carbon nanotubes
ATRA: All-*trans*-retinoic acid
SCF: Stem cell factor
VEGF: Vascular endothelial growth factor
COX2: Cyclooxygenase 2
EMT: Epithelial mesenchymal transition.
